# Typology of coastal urban vulnerability under rapid urbanization

**DOI:** 10.1371/journal.pone.0220936

**Published:** 2020-01-31

**Authors:** Till Sterzel, Matthias K. B. Lüdeke, Carsten Walther, Marcel T. Kok, Diana Sietz, Paul L. Lucas

**Affiliations:** 1 Climate-babel, Beelitz-Heilstätten, Germany; 2 Potsdam Institute for Climate Impact Research, Research Domain II–Climate Resilience, Telegraphenberg, Potsdam, Germany; 3 PBL Netherlands Environmental Assessment Agency, AH Bilthoven, The Netherlands; University of Lausanne, SWITZERLAND

## Abstract

Coastal areas are urbanizing at unprecedented rates, particularly in low- and middle-income countries. Combinations of long-standing and emerging problems in these urban areas generate vulnerability for human well-being and ecosystems alike. This baseline study provides a spatially explicit global systematization of these problems into typical urban vulnerability profiles for the year 2000 using largely sub-national data. Using 11 indicator datasets for urban expansion, urban population growth, marginalization of poor populations, government effectiveness, exposures and damages to climate-related extreme events, low-lying settlement, and wetlands prevalence, a cluster analysis reveals a global typology of seven clearly distinguishable clusters, or urban profiles of vulnerability. Each profile is characterized by a specific data-value combination of indicators representing mechanisms that generate vulnerability. Using 21 studies for testing the plausibility, we identify seven key profile-based vulnerabilities for urban populations, which are relevant in the context of global urbanization, expansion, and climate change. We show which urban coasts are similar in this regard. Sensitivity and exposure to extreme climate-related events, and government effectiveness, are the most important factors for the huge asymmetries of vulnerability between profiles. Against the background of underlying global trends we propose entry points for profile-based vulnerability reduction. The study provides a baseline for further pattern analysis in the rapidly urbanizing coastal fringe as data availability increases. We propose to explore socio-ecologically similar coastal urban areas as a basis for sharing experience and vulnerability-reducing measures among them.

## 1. Introduction

Urbanization is a defining phenomenon of our time [1]. Most urbanization takes place in the developing world, and cities are disproportionately located along rivers and coastlines [2]. 40% of the world’s population inhabit a narrow coastal band that takes up 7% of the Earth’s land area [3].

Urban development is radically changing coastal environments through unmanaged population increase [2], urban expansion [4], and resource demand [5]. Long-standing and emerging socio-economic and biophysical problems for coastal cities are compounding because of the magnitude and acceleration of transitions [6]. In rapidly growing cities in low- and middle-income countries (LICs and MICs) in particular, these problems risk to outpace the efforts to reduce vulnerability [7–9]: They increase exposures and sensitivity of urban populations and ecosystems [3] to multiple socio-ecological problems, and overstretch municipal management and planning capacity [10,11]. In this context we understand urbanization primarily as urban population increase implying different rates of areal extension. Annual urban population change rate of at least 2.25% denotes above-average, or rapid, urban population increase—of at least 25% from 1990 to 2000—according to the UN World Urbanization Prospects [12]. Garschagen & Romero-Lankao [13] point out that greater scientific attention is needed for the linkages between different components of vulnerability of urban populations. This could identify entry points to enhance the adaptive capacity at the city and national level, strengthen resilience for coastal cities under rapid growth, and facilitate learning in city networks.

Regional and local case-study literature shows that there are different types of vulnerability in urban coastal areas and the challenges outlined above are unevenly distributed geographically. For example, urban areas facing floods show significant variations in rates and magnitudes of urban expansion, which contribute to significant variations in exposure [14]. Despite the knowledge about these challenges and the vulnerability they generate for urban populations, there is a lack of integrated studies on a global scale that systematize the challenges for different types of cities at various locations.

The recent emergence of international city networks on the subject of climate change hints to shared interest of cities in collaborating and learning from each other to improve their adaptive capacity under rapid urbanization. Prominent examples are the C40 Cities Climate Leadership Group [15], the World Mayors Council on Climate Change (WMCCC) [16], and the Urban Climate Change Research Network UCCRN [17]. Besides local case-study knowledge this collaboration requires generic insights that acknowledge local specifics and still constitute a global picture of coastal vulnerability of urban populations. While a general scaling up of effective vulnerability-reducing measures is difficult [18], an informed indication of similar urban areas elsewhere would be an important step towards the successful transfer of measures and strategies.

So far, some studies analyze socio-ecological problems based on recent or projected future biophysical risks in a limited number of coastal cities or urban areas [3] [19][20] [6] [21].

Additionally, some regional [22] and global-level studies [14] [23] have also recently emerged, which focus on vulnerabilities in urban areas. Yet none focus on the interaction of socio-economic and biophysical problems related to rapid growth at the global coastal fringe.

Beyond these studies there is a need for identifying patterns among the global diversity of cities from which conclusions can be drawn for transforming urban areas to sustainability [24]. This paper aims to identify the first spatially-explicit typology of vulnerability of urban populations under rapid urbanization along the global coastal fringe with subnational spatial resolution (0.5° x 0.5°, or 30 arc minutes). For this area we present a near-recent systematization of typical causes of urban socio-ecological vulnerability under rapid urbanization (urban population increase greater than 2.25%, see Supporting Information) circa the year 2000 –the most recent reference year for all required dataset at the time of this analysis (see discussion in Section 4.7 for details).

We seek to answer the following research questions:

1. In how far do urban coasts share characteristic socio-ecological vulnerabilities to co-occurring socio-economic and biophysical problems under rapid urbanization?
2. How are the selected urban areas positioned to deal with and reduce these shared vulnerabilities?

For this purpose we applied a formalized method based on clustering [25,26]. This method has been successfully applied before to identify and interpret socio-ecological problems which similarly generate vulnerability in global drylands on regional and local scales [18,27–29].

## 2. Methods and data

Our method for systematizing how and where vulnerability is typically generated follows a method of vulnerability analysis on an intermediate level of complexity (of mechanisms and conditions) and spatial scale (allowing for global coverage) proposed by Kok et al. [25]. The method consists of four steps:

1. Defining the relevant and distinct socio-ecological system for vulnerability analysis
  We have defined rapidly urbanizing coastal areas as such a system in the introduction.
2. Identification of vulnerability creating mechanisms, and their key determinants and indicators
  Drawing from a multitude of case studies and meta-analyses of cases studies in the literature we list and briefly characterize well-documented mechanisms that have been found to typically generate vulnerability for urban populations in coastal urban areas. We identify key determinants of these vulnerability-creating mechanisms. Then we determine indicator datasets that render quantitative information on these key determinants of vulnerability. Consequently, the combination of these indicators can be interpreted in terms of the vulnerability-creating mechanisms.
3. Identification of vulnerability profiles and their spatial distribution
  We submit these indicators to a cluster analysis [26,30] to determine in how far typical indicator value combinations, which resemble vulnerability-creating mechanisms occur. Thereby the center of each resulting cluster signifies an urban vulnerability profile.
4. Urban vulnerability profiles: characteristics, interpretation and verification
  In this analytical step each urban vulnerability profile is described and interpreted. This is done in the light of the documented key determinants of the vulnerability-creating mechanisms. Thereby we analyze what drives vulnerability in each profile, explain differences between profiles, and test these results against local realities according to case studies and meta-analyses of case studies. We employ each profile’s spatial distribution, values for each indicator, and box plots to aid the analysis.

### Identification of vulnerability-generating mechanisms, key determinants, and indicators

Following a literature review we specified several mechanisms through which rapid coastal urbanization, i.e. rapid urban population increase, typically generates vulnerability.

Coastal cities are already disproportionately **exposed and sensitive to cyclones** [19,31] **and floods** [19,32]. Sections of most of the largest African coastal cities are currently at risk of flooding [33–35]. Examples of cities that are subject to increasing exposure and sensitivity to climate extremes include Dhaka (storm surges) [36], Sorsogon City, Philippines (taifuns) [37], Mumbai, Rio de Janeiro, and Shanghai (floods) [21]. The areas at risk are expected to significantly increase in the near term by **urban land expansion** [14] which is more rapid in low elevation coastal zones (defined as the contiguous area along the coast that is less than 10 metres above sea level) than in other places [4]. It causes large-scale land cover change in LICs and MICs [4,38], i.e. in countries that are generally experiencing much higher levels of urban expansion and population growth [39]. Unprecedented degradation and destruction of wetlands surrounding coastal urban agglomerations through increased demand for land [40] and subsequent encroachment [3,38,41] destroys important ecosystem functions and services for the urban and surrounding populations, in particular flood regulation [41–43]. This was shown in New Orleans, after the landfall of Hurricane Katrina in 2005 [44]. Depending on the specific ecosystem functions the wetlands provide for the urban population, their degradation has serious implications for urban livelihoods [45].

Rapid urbanization is increasing the vulnerabilities in urban populations by overstretching **municipal management and planning capacity** [6,10,11]. The **rapid population growth** from in-migration and internal population increase can overwhelm basic urban services, especially if municipal adaptive capacity is initially low. This overstretch occurs when new problems arise before long-standing ones have been dealt with [6]. Future climate change, for example, is expected to further stretch the management capacity in coastal areas [21,46,47] Overstretched management has been well documented in case studies, e.g. in Dhaka, Dar es Salaam [47] and Mumbai [21]. In the remainder of this article, the term, management’ comprises both management and planning.

Rapid population increases are often absorbed into the urban fabric through an increase in densely populated informal settlements [11]. The growth in informal settlements is largely driven by poverty and **marginalisation of poor populations** in and around megacities in developing countries [41,48] and is frequently underestimated [49]. Informal settlements have less capacity to deal with shocks, e.g. climate-related extremes [43,50,51] such as tropical cyclones and floods [52], and less capacity to deal with the ensuing negative impacts [53]. This particularly applies when poor management, and low building and infrastructure quality coincide with densely populated areas [11]. Marginalization of the poor has been observed inter alia in numerous coastal cities in India [54] and in Iliolo City in the Philippines [55]. Under inadequate urban management and unchecked growth, informal settlements commonly encroach more risk-prone areas where flood and cyclone exposure is high [56–58]. These areas are avoided by wealthier populations [11]. This leads to an increase in the number of vulnerable populations, population density, and low-quality buildings in floodplains. Informal settlement development in exposed areas has been observed in cities such as Lagos [34], Mumbai [59], Esmeraldas, Ecuador [60], and many other large African coastal cities [48].

**Fig 1:**
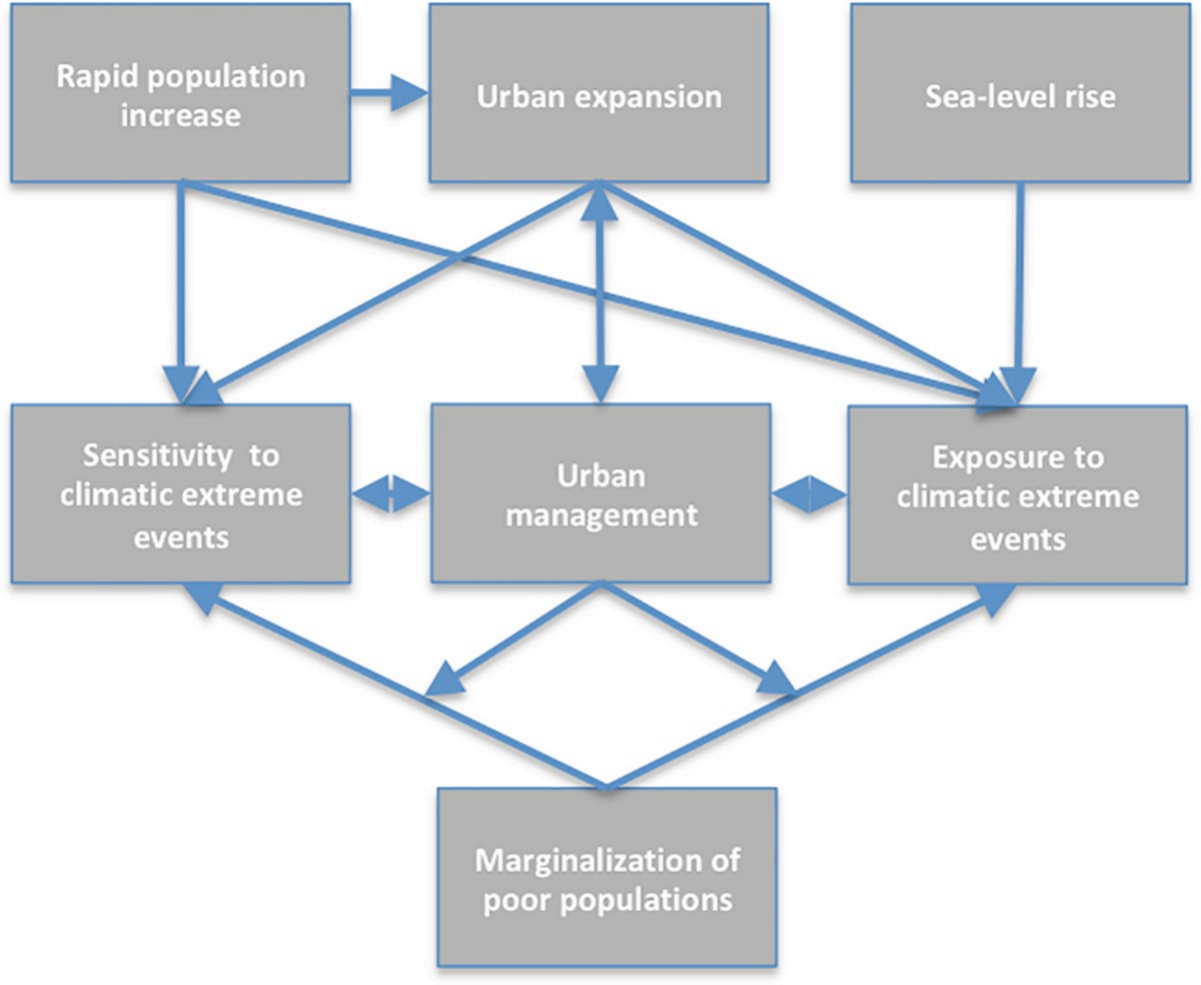
Key determinants and their interactions, describing socio-ecological vulnerability under rapid coastal urbanization. Arrows indicate the direction of influence.

**Fig 2:**
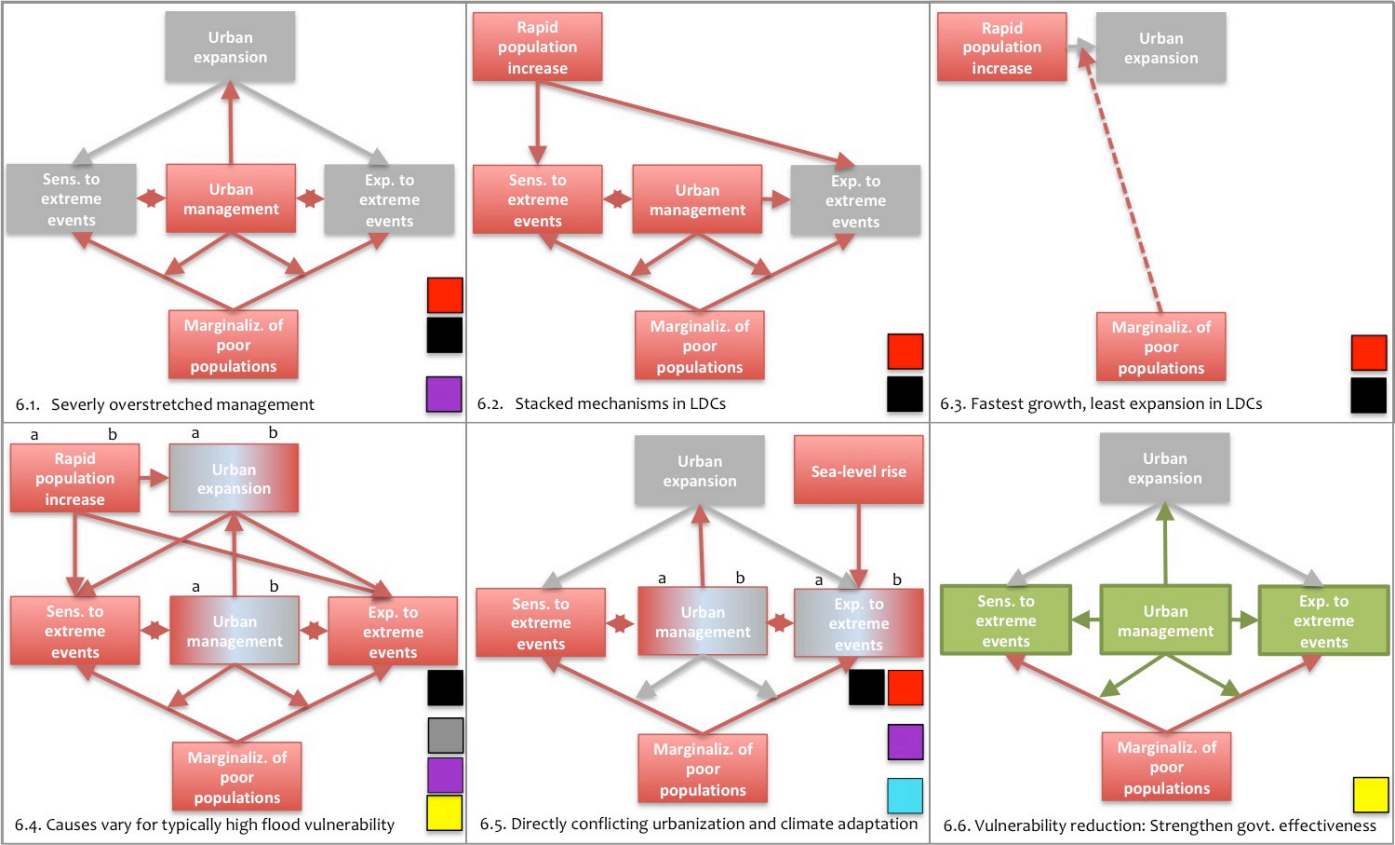
Six typical configurations of socio-ecological vulnerability of urban populations under rapid coastal urbanization. Based on Fig 1 (key determinants of vulnerability and how they influence each other) each diagram is a typical and particularly problematic configuration. Red boxes and arrows signify particularly problematic situations and influences based on indicator values, grey boxes signify less problematic situations and influences. Green boxes and arrows refer to entry points for vulnerability reduction. The number of each diagram refers to a corresponding subsection in the Discussion section. The colored boxes in the lower right hand corners indicate which urban vulnerability profiles each diagram applies to. LDCs stands for Least Developed Countries.

Although future estimates of the additional number of people at risk from coastal flooding vary widely [61], all indicate a considerable increase due to surging populations in low-lying areas and to sea-level rise. This aspect becomes even more serious as the projections for future sea-level rise are adjusted upwards recently (e.g. [62]). The ongoing superimposition of **sea-level rise** on current flood levels increases the level of vulnerability in coastal cities to climate extremes [63]. This poses a major challenge for coastal management in terms of adapting to both rising storm surge levels and rising flood levels [8,64]. We summarize the major findings of the above-cited literature on urban socio-ecological vulnerability by seven key determinants and their interactions in Fig 1. This figure is also a template for all diagrams in Fig 2 in the discussion section, which each displays a typical and particularly problematic interaction of some of the key determinants.

As depicted in Fig 1 **rapid urban population increase** typically increases the **exposure and sensitivity** of urban populations **to climatic extreme events** under overstretched or unwise **urban management**. The increase in exposure is also driven by unmanaged **urban expansion** into risk prone areas, and by **sea-level rise**. The increase in exposure and sensitivity is also driven by the **marginalization of poor populations** into risk prone low-lying areas and dense informal settlement development, and by degradation of flood regulating ecosystems such as wetlands through unchecked **urban expansion**, when **urban management** cannot effectively regulate development.

We identified 11 indicators with global coverage to render quantitative information on the key determinants (Table 1, [102]). We did not impose a hypothesized predefined relationship between the indicators, following Kok et al. [25]. Instead, we (inductively) explored the structure in the data-space to obtain insights into typical vulnerability patterns. In Table 1 each indicator is attributed to one of the three vulnerability components commonly used in frameworks for vulnerability analysis in the context of global environmental change: exposure, sensitivity and adaptive capacity [65–67]. Thereby we understand ‘damage’, e.g. from floods, as ‘exposure times sensitivity’.

**Table 1:** Indicators and indicator datasets used, including their assignment to vulnerability components, the key determinants they are related to, and the level of spatial data aggregation.

Key determinant	Indicator	Vulnerability component	Indicator dataset	Data source	Processing in R	Spatial resolution after aggregation	Original spatial resolution
Rapid population increase	Rapid urban population increase	Exposure	Changes in urban population from 1990 to 2000 in percent of 1990	Hyde 3.1 [68]		0.5°x0.5°	5 arc min.
Urban expansion	Urban expansion		Changes in urbanized area from 1990 to 2000 in percent of 1990	Hyde 3.1 [68]		0.5°x0.5°	5 arc min.
Urban management	Government effectiveness	Adaptive capacity	Government effectiveness	Worldwide Governance indicators (WGI) [69]		National	National
Marginalisation of poor populations	Average per capita income		Per capita GDP	World Development Indicators (WDI), [70], data gaps supplemented with UNSTAT [71]		National	National
	Slum population level	Sensitivity	Slum population in 2000 in percentage of the urban population	UN-HABITAT [72]		National	National
Sensitivity to/ damage from climatic extreme events	Wetlands prevalence		Combination of prevalence of key wetlands, and the percentage of those that immediately surround urban areas	Global dataset on wetlands, lakes, and reservoirs [73], urban extent data [74]		0.5°x0.5°	Polygon vectors (wetlands); 2.5 arc min.
	Cyclone damage	Damage	Fatalities per year from cyclones	Natural disaster hotspots [75]	File cycl_mean.txt missing values set to 0; aggregated by factor 12 in AT3-makedata_revival.R	0.5°x0.5°	2.5 arc min.
	Flood damage		Fatalities per year from floods	Natural disaster hotspots [75]	File flood_mean.txt missing value set to 0; aggregated in R by factor 12 in AT3-makedata_revival.R	0.5°x0.5°	2.5 arc min.
Exposure to climatic extreme events	Cyclone occurrence	Exposure	Average relative frequency and distribution of cyclones	Natural disaster hotspots [75]	File cyclone_freq_mean.asc missing value set to 0 from cyclone_freq_refined.rar June 2009 folder contains shp-files aggregated in R by factor 12 in AT3-makedata.R	0.5°x0.5°	2.5 arc min.
	Flood occurrence		Average relative frequency and distribution of high floods	Natural disaster hotspots [75]	File flood_freq_mean.asc missing value set to 0 from flood_freq_refined.rar; aggregated in R by factor 12 in AT3-makedata.R	0.5°x0.5°	2.5 arc min.
Sea-level rise	Low-lying settlement		Total urban population currently living 2m or less above sea level; calculated using the digital elevation model SRTM v4.1	Digital elevation model SRTM v4.1 [76] and urban population data [68]		0.5°x0.5°	3 arc sec. (SRTM); 5 arc min.

The reasons for choosing each dataset, and details on data resolution and data treatment, are provided in the Supporting Information. A spatial resolution of 0.5°lon x 0.5°lat (30 arc minutes), which is compatible with the integrated assessment model IMAGE [77], was chosen for the indicators: The analysis presented in this paper focused on eliciting the most recent patterns of vulnerability based on a common time frame for all the data required. This is extensively discussed in the section “Discussion of the method”. However, there is a clear interest in how the patterns evolve over time, especially under future climate change. Integrated assessment models such as IMAGE can provide consistent data for future projections for this analysis, taking into account different assumptions and uncertainties. Using data projections from the IMAGE model Lüdeke et al. [30] already analyzed projected changes in patterns of smallholder vulnerability to global environmental change in global drylands.

Many of the datasets used for the indicators were on a much finer scale, such as 3 arc seconds for [76], 2.5 arc minutes for [75], and 5 arc minutes for [68]. These datasets were aggregated to 30 arc minutes for the analysis.

### Identification of vulnerability profiles and their spatial distribution

We applied the established cluster analysis method k-means to integrate the 11 datasets and to identify typical combinations in the data structure [26,30]. This method is suitable for large datasets and generates rather compact clusters [78] which fits our objective to identify typical vulnerability profiles, represented by the cluster center. The optimal number of clusters in the data set was identified by applying a consistency measure (see, e.g., 15). After multiple repetition of the clustering process and the corresponding allocation of grid cells to clusters, this measure analyzes the stability of the results depending on the cluster number. If the pre-given number of clusters fits the underlying structure in the analyzed data set the results become more stable (see Supporting Information for further details on the cluster analysis and consistency measure (including S1 Fig)).

The Fraiman measure was employed to investigate the importance of each indicator in shaping the clusters. With this measure we were able to determine how sensitive the cluster partition was to each single indicator by fixing it at its mean-value (so-called ‘blinding’ of indicators), and comparing ‘blinded’ runs of the clustering with the runs that included all indicators without blinding [79].

## 3. Results: Urban vulnerability profiles

**Fig 3:**
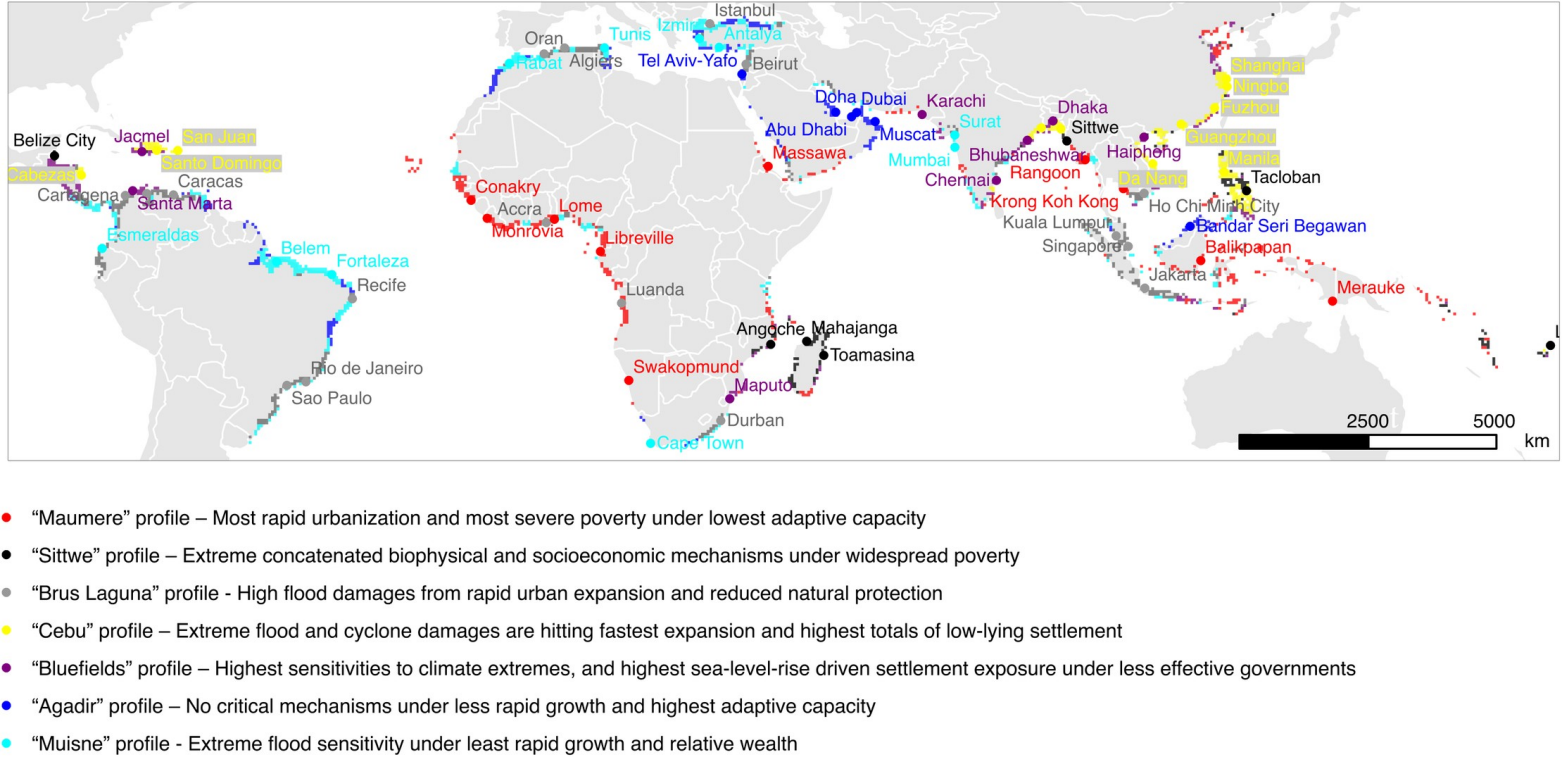
Spatial distribution of the seven urban vulnerability profiles under rapid coastal urbanization, and examples of cities located in these profiles. See Table 2 for a short description of the respective profiles.

Our analysis separates the rapidly urbanizing coastal fringe into seven robust and distinct vulnerability profiles. Eighty-four out of a total of 196 countries (43%), and out of 153 countries with a coastline (55%), are experiencing rapid coastal urbanization. Since the overwhelming majority of urban coasts in -income countries were excluded not a priori, but rather by the threshold we used for above-average urban population increase rate, the geographical distribution of the seven profiles confirms that rapid urban growth is overwhelmingly a phenomenon in LICs and MICs (Fig 3). Table 2 summarizes key characteristics for vulnerability, examples of cities, geographical distribution, and population statistics in each of the profiles.

**Table 2:** Vulnerability profiles—Key characteristics, city examples, and geographic distribution.

Profile group and profile	Key vulnerability characteristics	Examples of countries and geographical regions	Examples of cities located in profile	No. of coun- tries	% of global urban pop.	Urban pop. (m)	% of typology (No. of grid cells)
**I: Fastest population increase and pronounced poverty under overstretched management**				** **	** **		
**I.1 "Maumere” profile**	Most rapid urbanization and most severe poverty under lowest adaptive capacity	Most prevalent in Least Developed Countries, Sub-Saharan Western African and equatorial coasts; Yemen, Eritrea, Pakistan, Myanmar, Eastern Indonesia, Solomon Islands, Vanuatu; non-existent in Latin America	Maumere (Indonesia), Rangoon, Krong Koh Kong, Balikpapan, Monrovia, Conakry, Lomé, Libreville, Swakopmund, Massawa	41	1.8	51.4	18 (366)
**I.2 "Sittwe" profile**	Multiple severe biophysical and socio-economic problems under widespread poverty	Most prevalent in cyclone-prone Madagascar and Mozambique; Myanmar, Central Philippines, Belize; absent in South America	Small and middle-sized cities such as Sittwe (Myanmar), Toamasina, Mahajanga, Quelimane, Angoche, Sittwe, Tacloban, Labasa, Belize City	16	0.5	14.9	6.9 (140)
**II: Rapid population increase and most rapid expansion intensify high flood damages under moderate adaptive capacity**							
**II.1** **"Brus Laguna” profile**	High flood damages from rapid urban expansion and reduced natural protection	Southern Brazil, Ecuador, Peru, Columbia, Venezuela, Algeria, South Africa, Lebanon, NW India, SE India, Mekong Delta, Java, Sumatra, Southern Malaysia	Brus Laguna (Honduras), Rio de Janeiro, Sao Paolo, Maracaibo, Caracas, lgiers, Istanbul, Oran, Durban, Accra, Luanda, Beirut, Ho Chi Minh City, Jakarta, Kuala Lumpur	39	7.3	209.6	21 (427)
**II.2 "Cebu" profile**	Extreme flood and cyclone damages are hitting fastest expansion and highest totals of low-lying settlement	Subtropical coasts affected by tropical cyclones in Asia and Central America, majority of The Philippines, China, Vietnam, and Bangladesh; India (Bay of Bengal); nonexistent in Africa, virtually nonexistent in the southern hemisphere	Cebu (Philippines), Manila, Guangzhou, Shanghai, Fuzhou, Chittagong, Da Nang, Kolkata, Santo Domingo, San Juan, Puerto Cabezas	12	5.2	149.1	11.6 (237)
**II.3** **"Bluefields" profile**	High damages and moderate occurrence of climate extremes: most severe climate-change vulnerability	Subtropical coasts under less exposure to tropical cyclones in Asia (Southern Philippines, Indian—Bay of Bengal) and Central America (Belize, Guatemala, Honduras), The Caribbean (Southern Haiti and the Dominican Republic)	Bluefields (Nicaragua), Dhaka and Khulna, Chennai, Karachi, Maputo, Bhubaneshwar, Haiphong, Jacmel, Santa Marta (Columbia)	23	2.7	78.3	10.6 (215)
**III: Few and less severe problems under slower population increase and high adaptive capacity**							
**III.1 "Agadir" profile**	No severe problems under less rapid population increase and highest adaptive capacity	High-income countries on the Arabian Peninsula, Morocco, Tunisia, Guyana, Central Brazil, Turkey, Israel, Brunei, Malaysia	Agadir (Morocco), Abu Dhabi, Dubai, Doha, Muscat, Tel-Aviv, Bandar Seri Begawan (Brunei)	23	1	30.1	11 (223)
**III.2 "Muisne" profile**	Extreme flood sensitivity under relative wealth and least rapid population increase	Prevalent in South America, (e.g. Panamá, El Salvador, and NE Brazil) Magreb countries (e.g. Morocco, and Tunisia); Turkey, South Africa, West-Indian coast	Muisne (Ecuador), Esmeraldas, Natal, Fortaleza, Belém, Rabat, Tunis, Cape Town, Izmir, Antalya, Mumbai, Surat	43	3.6	102.9	21 (428)
**SUM**					26.6	209.6	2036

**Fig 4:**
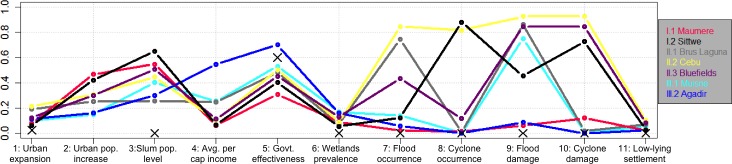
Vulnerability profiles and their average indicator values. The colored dots show the indicator values of the respective cluster centers. ‘X’ shows where the value is zero for each indicator. The indicator values are normalized between 0 and 1 using the minimum and maximum values for the different indicators. The colors are identical to those used in Fig 3 to depict the geographical distributions of the profiles. Each profile is given a name of a characteristic city located in it to aid the reader. Each indicator is numbered from V1 to V11 for easier comparison to Fig 5.

**Fig 5:**
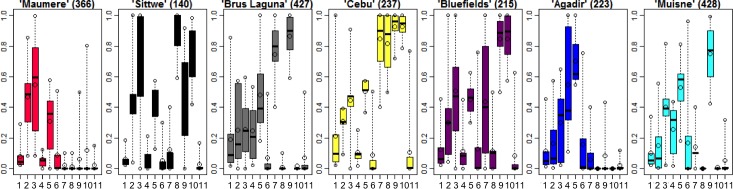
Box plots of the vulnerability profiles. They show the variation in indicator values (all indicator values are between 0 and 1) in each profile. The order of the indicators is identical to Fig 4. The boxes present the 25–75 percentile range of the indicator values; the circles at the end of the dotted lines indicate the 5- and 95-percentile, while the larger circle between them indicates the arithmetic mean; the black band in the box indicates the median value. The number of grid cells in a profile is indicated at the top of each frame.

**Fig 6:**
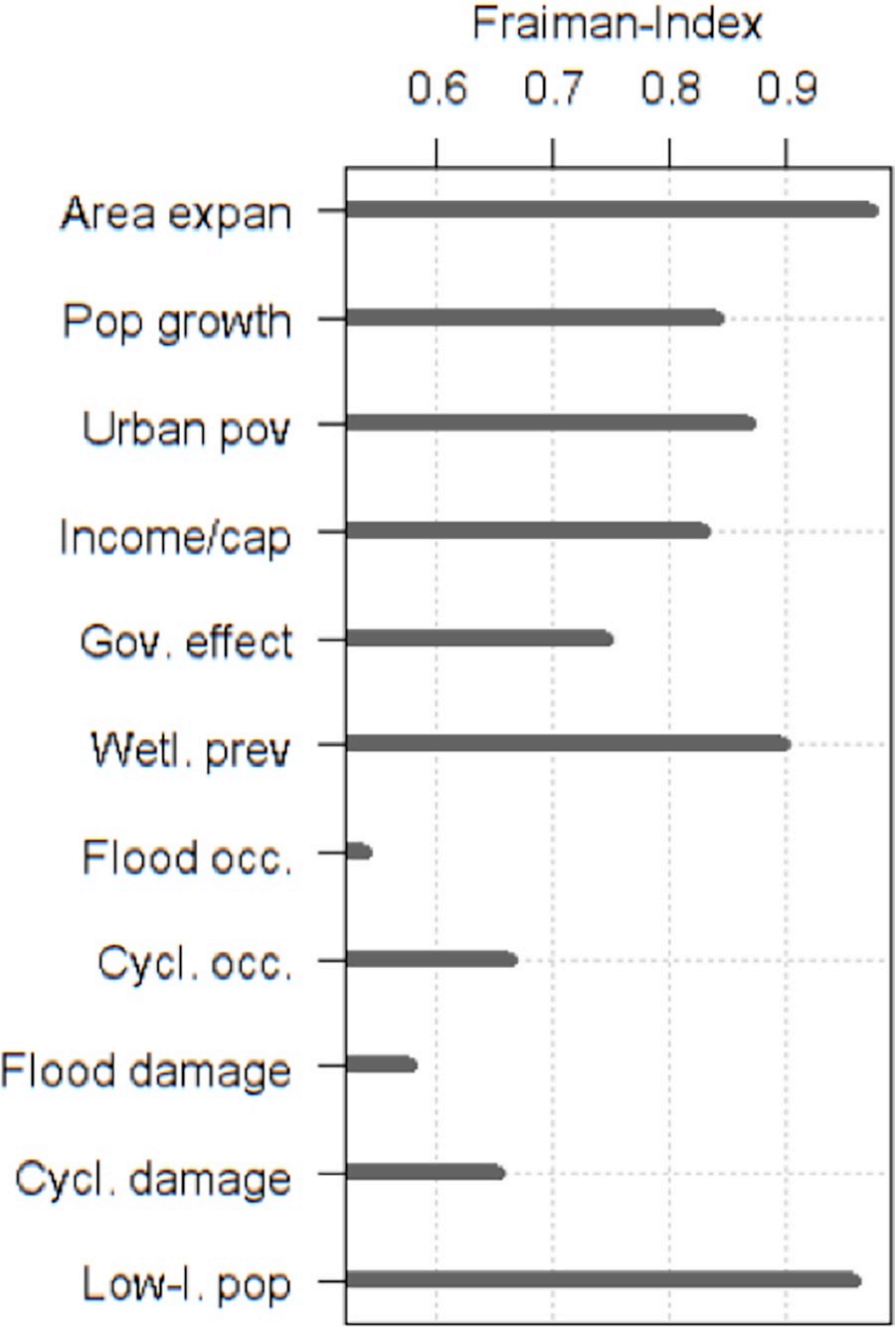
The Fraiman measure for each indicator. The values are between 0 and 1 and express the relative importance of each indicator in separating the clusters. The smaller the value, the more important the indicator is. The value shows the percentage of grid cells identically assigned when the corresponding indicator is blinded. Indicator names are abbreviated—Area expan: Urban expansion; Pop growth: (Rapid) urban population increase; Urban pov: Slum population level; Income/cap: Average per capita income; Gov. effect: Government effectiveness; Wetl. Prev: Wetlands prevalence; Flood occ.: Flood occurrence; Cycl. occ.: Cyclone occurrence; Flood damage: ibid.; Cycl. damage: ibid.; Low-l. pop: Low-lying population.

Closer inspection of differences in indicator values between profiles (Fig 4) reveals three groups of profiles (Fig 5 shows the box plots of all profiles). These groups separate significantly in regards to the rapid urban population-increase indicator while the profiles within each group are characterized by similar values for most of their indicators. Group I shows the most rapid population increase (2 profiles), group II a rapid increase (3 profiles), and group III a less rapid increase (2 profiles). The two profiles within group I are significantly distinguished by cyclone occurrence and damage, and less so by flood occurrence and damage. Profiles within group II are significantly distinguished by wetland prevalence, and specific combinations of cyclone damage and occurrence. Flood damage is the distinguishing indicator in group III. The chosen grouping minimizes the mean absolute indicator difference between the profiles within each group compared to the inter-group differences. Furthermore, four of the five indicators for intra-group distinctions are the most influential indicators for cluster identification (Fig 6, low Fraiman Index).

In the subsequent sections the seven profiles are characterized using their specific indicator value combinations from Fig 4 and the key determinants and the indicators relate to (Table 1). Each profile is named after a particularly representative city located in it to aid the reader. Each characteristic city is a particularly close match to its profile’s average indicator values. (Each one is located in one of the 20 grid cells with the smallest Euclidean distance to the corresponding cluster center). The 21 studies used for validating the profiles are listed in the Supporting Information.

### Group I: Fastest population increase and pronounced poverty under overstretched management

The red and black profiles in Fig 4 are characterized by extreme (i.e. severe) manifestations of problems: the most rapidly increasing coastal populations go together with the highest slum-population levels of all profiles (i.e. the highest averages for these two indicators). The co-occurrence of the lowest increase rates in urban expansion hint at relatively dense informal settlement development. Judging by the lowest values for indicators of adaptive capacity—government effectiveness and income–urban management in this group is severely overstretched by such rapid growth and urban poverty. This indicates that the urban areas characterized by these profiles are in an extremely challenging position to reduce vulnerability. The two profiles significantly differ in cyclone occurrence and–less significantly—in damages from climate extremes.

#### Profile I.1: “Maumere” profile (red)–Most rapid urbanization and most severe poverty under lowest adaptive capacity

The red profile is named after the city of Maumere on Flores, Indonesia. Occurrence of and damages from climate extremes are characteristically low (no robust statements on recent sensitivity towards climate extremes are possible). They show the smallest indicator values and spread in the box plot, signifying a clear difference to profile I.2 (Fig 5). The Maumere profile is spatially restricted to Least Developed Countries (LDCs). For these countries the combination of high slum population levels, the most rapid urbanization and overstretched urban management is in line with general findings by Garschagen and Romero-Lankao [13] and Cohen [80] on the national level.

#### Profile I.2: “Sittwe” profile (black)–Multiple severe biophysical and socio-economic problems under widespread poverty

The relatively rare black profile is named after the city of Sittwe in Myanmar. In contrast to the Maumere profile these urban areas suffer from higher flood and cyclone damages. In fact, the African cities in this profile (Table 2) are considered the most at risk to cyclones in all of Africa [81]. These damages exacerbate the severe socio-economic problem structure of very high urban poverty, slum levels, and management overstretch, which is specific to group I. Exactly this combination of vulnerability-generating mechanisms is illustrated in the case study of Sorsogon City [37].

Compared to the Maumere profile, relating the larger damages from floods with the low flood occurrence yields relatively high flood sensitivity. This can be explained by the lowest urban expansion rate of any profile, which indicates an absorption of burgeoning population increase through marginalization of the poor in dense informal settlements. This worsens existing risks for poor households which is a well-developed argument in developing countries [6,82].

A case study on the preparedness, response and reconstruction activities in Tacloban/Phillipines [83] after the Typhoon Haiyan illustrates the management-overstretch problem in this cluster and gives some hints for improvement. Despite several promising national legislative and institutional activities, critical gaps in managing disaster responses and recovery across tiers of government from national to city and local levels down to communities, households, and individuals occurred. So here the main goal should be “to transform national policies into content aligned with the realities of provinces, city governments, communities, and households while maintaining its consistency, completeness, and integrity with national policies in terms of visions and goals” [83].

### Group II: Rapid population increase and most rapid expansion intensify high flood damages under moderate adaptive capacity

The three group II profiles (grey, yellow, purple) display the highest flood occurrences and damages, the highest totals of low-lying settlement, the fastest urban expansion rates, and relatively high wetland prevalence. In mechanistic terms, this indicates common urban expansion into flood-prone low-lying areas. The product of expansion rate and wetland prevalence is similar for the three profiles, indicating a high group-typical endangerment of the remaining natural flood protection. The three profiles subsumed under group II differ significantly in cyclone occurrence and damages, and differ to some extent in slum population levels and income.

#### Profile II.1.: “Brus Laguna” profile (grey)—High flood damages from rapid urban expansion and reduced natural protection

The grey profile is named after the city of Brus Laguna in Honduras on the Gulf of Mexico. It is a particularly distinct, robust structure in the data space, as it is discernable in clusterings with predetermined numbers of cluster from two to six as well. There is essentially no tropical cyclone activity (lowest occurrence and damages of all profiles). The lowest slum-population levels and highest income in group II indicate high adaptive capacity to flooding, and low socio-economic disparities, for this group. However, in mechanistic terms, high flood occurrence, fast-paced urban expansion, reduced natural flood protection, and relatively high totals of low-lying settlement translate into high flood damages. This combination has been observed in a case study in Rio de Janeiro (which belongs to this profile) on vulnerability to current climate hazards [21]. The socio-economic situation in cities such as Rio, Durban, or Kuala Lumpur is clearly more favorable than in the related Cebu and Bluefields profiles. This and relatively high governance effectiveness allow for measures to reduce vulnerability as e.g. realized in Ho Chi Minh City [84]. Here the challenges of urban flooding caused by insufficient drainage capacity under heavy rainfalls, sea-level rise and expansion of the city into low-lying marshlands are successfully tackled by an integrated land and water systems approach. Multi-scale flood protection, smart urban density, improved drainage and storage systems (e.g. by urban parks), and enforcing new developments to be elevated 2 meters above mean sea level are put in place by a fully committed city administration.

#### Profile II.2: “Cebu” profile (yellow)–Extreme flood and cyclone damages are hitting fastest expansion and highest totals of low-lying settlement

The yellow profile is named after the city of Cebu in The Philippines. The narrow indicator value distributions in the box plot signify a particularly distinct profile in the data space. In addition to the typical flood problems of group II, the very high occurrence of and damages from cyclones distinguish the Cebu profile. It is characterized by a severe confluence of problems: In mechanistic terms, the fastest urban expansion is taking place under highest overall damages from climate extremes and highest totals of low-lying settlement. A low prevalence of wetlands suggests that rapid urban expansion may be endangering the few existing natural flood-regulating ecosystems. A global ranking of population numbers in large port cities which are exposed to current coastal flooding [19,31] shows that three of the four highest ranked cities are located in this profile (Shanghai, Guangzhou, and Kolkata).

In regards to adaptive capacity, the high level of government effectiveness is comparable to profiles with much higher average income, less poverty, and lower occurrence of climate extremes. On the other hand, moderate slum population levels, and average income, indicate relatively low capacity to reduce the very high flooding and cyclone damages. Taking city case studies located in this profile into account, the relatively high marginalization of the poor, low income, and rapid growth suggest that the low-lying settlement areas are also inhabited by marginalized informal settlements: In Manila, for example, informal settlements at risk to coastal flooding make up 35% of the population [85]. This combination of problems is also documented in Shanghai [21]. Regarding vulnerable informal settlements, in Manila there are initial steps to provide in situ resettlement through construction upgrading and capacity-building in disaster preparedness for informal communities. Integration of the informal economy by recognizing its value contributions to formal supply and service chains is considered. The institutionalization of green building codes, solid waste management and recycling programs by several local governments in the metropolis is now under way [83] (case study 11.B)

Present flood damages in the Cebu profile show a high vulnerability to sea-level rise owing to its superimposition on coastal floods and storm surge levels. Hanson et al. [19] and Nicholls et al. [31] find that other cities in this profile, i.e. Shanghai, Guangzhou, Kolkata, Chittagong, and Ningbo, will be among the cities most exposed to coastal flooding in the 2070s because of sea-level rise and storm surge.

#### Profile II.3: “Bluefields” profile (purple)–High damages and moderate occurrence of climate extremes: most severe climate-change vulnerability

The Bluefields profile is named after the city of Bluefields in Nicaragua, and is prevalent in subtropical coasts with cyclone exposure in Asia, Central America, and The Caribbean. Differential analysis of the Bluefields and the Cebu profile reveals the most important distinguishing feature of this profile: Despite the lower occurrence of floods and cyclones, damages from climate extremes are similarly high, resulting in the highest sensitivities in group II. The higher adaptive capacity (Bruce Laguna & Cebu) and less climate extremes (Cebu-profile not hit by cyclones) might explain this difference.

The evidently overstretched management in this profile is illustrated by a case study in Chennai: Uncontrolled urban expansion as well as blockage and encroachment of natural drainage systems have increased coastal and riverine flooding. The flooding overwhelms a lacking flood-control response and the drainage systems [86]. Under these circumstances, relatively high wetlands prevalence alone does not significantly reduce high flood- and cyclone-related sensitivity. The profile’s extreme sensitivity to floods and storm surges has also been observed in Dhaka [8] and Maputo [48]: Even moderate flooding events have largely affected the urban poor. In conclusion, the urban areas in the Bluefields profile appear to be in the weakest position to deal with climate extremes and future sea-level rise.

Participatory action plan development in Maputo, analyzing the causes and impacts of flooding and potential future impacts and solutions shows a way to decrease vulnerability of poor neighborhoods even under the unfavorable conditions of this cluster [83] (case study 6.5). Community’s proposals to mitigate flood impacts like loss of access, damage to property and vector-borne diseases were based on self-organization (e.g. cleaning and maintaining drainage channels, waste separation and composting) but got additional support by local businesses, the water utility and the Ministry for Environmental Coordination.

### Group III: Few and less severe problems under slower population increase and high adaptive capacity

Group III profiles are characterized by the highest average income and government effectiveness, relatively low marginalization, and low occurrence of climate extremes. In mechanistic terms, the dark and light blue profiles are under the least pressure, and most favorably positioned to further reduce the few discernible problems: Urban management does not appear to be overstretched, and is subject to handling the “slowest” population increase rates. The two profiles subsumed under group III differ significantly in flood sensitivity, and less significantly in adaptive capacity.

#### Profile III.1: “Agadir” profile (dark blue)–No severe problems under less rapid population increase and highest adaptive capacity

The dark blue profile is named after the city of Agadir in Morocco, and is prevalent in MICs and high-income countries (HICs). Most effective government and highest income indicate a significantly better adaptive capacity than in any other profile. At the same time, cyclone and flood occurrence and damages alike are amongst the lowest in the entire fast-growing coastal fringe, leading to the lowest overall vulnerability to climate-related extremes. Therefore this profile starkly contrasts with the fast-growing and managerially overstretched profiles in LICs.

In mechanistic terms flood occurrence can increase in the future through a combination of continued rapid urban expansion into wetlands (and other low-lying areas), and sea-level rise. This combination of vulnerability-generating mechanisms has been suggested as a threat to unplanned rapid urbanization in coastal cities on the Arabian Peninsula [87]. In addition, the advantageous conditions to adapt to increasing flood exposure may lead to ignoring climate change adaptation requirements. This lack of precaution has been documented in the analysis of urban planning regulations in the Arab region [88]. On the other hand, the Masdar project (Abu Dhabi) might be acknowledged as a positive counter example [83] (case study 5.4) with regard to urban planning under expected climate change.

#### Profile III.2: “Muisne” profile (light blue)—Extreme flood sensitivity under relative wealth and least rapid population increase

The light blue profile is named after the city of Muisne in Ecuador, and is prevalent in MICs. In mechanistic terms, all vulnerability-generating problems are linked to high flood sensitivity. What distinguishes this profile from the wealthier Agadir profile and other profiles is the high flood damages under low flood occurrence in spite of relatively high average income and government effectiveness. The profile-based explanations for such flood sensitivity are a) marginalization of the poor in flood-prone areas under conditions of great socio-economic disparities, and b) a high differential impact of floods. This has been documented in Esmeraldas, Ecuador [60], as well as in cities such as Cape Town and Mumbai (cities with pronounced social disparities), where informal low-lying settlements lack drainage infrastructure [21,89,90]. Under these circumstances the high wetlands prevalence alone is insufficient for reducing major flood sensitivity. Regarding pluvial floods, an early warning system established in Surat, India [83] (case study 3.D) is a positive example which reduced damages significantly. As the upstream catchment area spans three states, including a large dam, cross-government bodies were established which share weather forecasts, hydraulic model results and potential damage assessments. The result is the damage minimizing management of the large upstream dam and an increase of advance warning time from one to four days.

## 4. Discussion

By interpreting urban vulnerability profiles in the light of the documented key determinants, and validating the profiles using local case studies, typical and particularly problematic interplays of key determinants of vulnerability become evident. This discussion focuses on these interplays: six key vulnerabilities for urban populations, which have particular relevance in the context of urbanization, expansion, and climate change.

Rapid urbanization can also offer opportunities for reducing vulnerability [13,91], and can play an important role in economic development [92]. Therefore, we also explore profile-specific entry points for reducing these typical vulnerabilities.

### 4.1. Severely overstretched urban management (Group I and Bluefields profiles)

Vulnerabilities to global change can outpace vulnerability reduction in rapidly growing LICs and MICs [7,9]. If overstretched management–indicated by ineffective government under low average income under a multitude of severe problems—is a proxy of this risk, then our study suggests that vulnerability reduction is being, or risks being, outpaced in over 50 (60%) out of 84 countries exhibiting rapid coastal urbanization (Fig 2, 2.1.). The three profiles that show the clearest signs of management overstretch (Sittwe, Maumere, and Bluefields profiles) account for over a third (36%) of all rapidly urbanizing coasts, and 145m people (5% of the global urban population) in 57 countries.

### 4.2. Vulnerability-generating mechanisms are dramatically stacked against coastal urban areas in LDCs (Group I profiles)

Inspection of all profiles shows that income and government effectiveness decrease with the rate of urban population growth, while urban poverty increases. The urban areas in group 1 (red Maumere and black Sittwe profile) exhibit both characteristics in extremis: They are simultaneously subject to the lowest income, most ineffective governments, most prevalent poverty, and world’s most rapid coastal population growth rates (Fig 2, 2.2.). This makes is clear that vulnerability-generating mechanisms are dramatically stacked against the fastest-growing and most impoverished coastal urban areas in the world, which at the same time have the least adaptive capacity to reduce vulnerability. This applies to many LDCs along Western and Eastern African tropical coasts, Yemen, Pakistan, Myanmar, the Eastern Indonesian Archipelago, Solomon Islands, and Vanuatu (Maumere profile). In the Sittwe profile the situation is additionally aggravated by high levels of vulnerability to climatic extreme events: This means that urban areas in Madagascar, northern Mozambique, Small Island Developing States, Myanmar, and parts of the Philippines are subject to a “worst case reality” of well documented drivers of coastal urban vulnerability. In these areas hydro-meteorological climate extremes are not stopping rapid urban population increase. Klose and Webersik [93,94] have shown that this is the case for population development and tropical storms in Haiti.

### 4.3. LDCs are the locus of the fastest but least expansive urbanization (Group I profiles)

Unsurprisingly, urban areas in all rapidly urbanizing coastal fringes are expanding. However, coastal urban population growth is most rapid where increase rates in urban area are slowest, i.e. in the red Maumere and black Sittwe profiles. The severe levels of slum population in these profiles suggest that the swell of inhabitants is largely absorbed on limited area by existing informal settlements and the erection of new ones (Fig 2, 2.3.). According to literature urban areas are generally increasing at twice their population growth rates [39], and population density is generally stagnating or in decline [4,39]. On the other hand, none of the 30 representative cities Angel et al.’s findings are based on are located in the Maumere or Sittwe profile. Therefore, further investigation of population density change in these urban areas could nuance the ratio between area and population growth rates on the extreme end of the urban spectrum, i.e. for the fastest growing cities with high slum population levels.

### 4.4. High flood vulnerability is typical, yet a result of different combinations of causes (Sittwe and Group II profiles)

In the past, flood vulnerability in urban areas has been largely driven by socio-economic mechanisms: Noted causes include marginalization of the poor, ecosystem degradation, and poorly governed rapid urbanization [19,51,54,95]. By indicating each of these causes, as well as flood occurrence, damages, and adaptive capacity, this study shows two things: that severe values of these causes are typically combined to generate high flood vulnerability, and in which coastal fringes they co-occur.

For one, the noted causes co-occur in extremis in the impoverished black Sittwe profile in LDCs, as discussed in 4.2 (Fig 2, 2.4a). These causes translate into large damages from floods, cyclones, or both. The causes also co-occur in all profiles of group 2 (grey Brus La Laguna, yellow Cebu, and purple Bluefields profiles). Here, however, high flood vulnerability is better explained by severe manifestations of different causes, namely the most rapid urban expansion rate and highest total of low-lying settlements (Fig 2, 2.4b). This co-occurrence supports the finding that expansion is more rapid in the low elevation coastal zone [4]. In these profiles, we conclude that flood exposure is driven by large-scale flood-prone expansion into low-lying areas and rapid population growth. Flood sensitivity is driven by massive loss of wetlands (Fig 2, 2.4.). On the one hand, this causal combination can lead to particularly high flood vulnerability under low government effectiveness (Bluefields profile). On the other hand, the same causal combination leads to lower flood vulnerability under more effective government (Cebu profile). This is noteworthy, because in this profile occurrence of and damages from floods *and* cyclones are extremely high, and its low-lying areas along the subtropical coasts in Asia and Central America, the majority of The Philippines, China, Vietnam, Bangladesh and India (Bay of Bengal) are heavily populated.

### 4.5. Patterns of most rapid coastal development are in direct conflict with current and future climate adaptation (Bluefields, Muisne and Group I profiles)

Urban areas with the most rapid growth rates for population and urban expansion, respectively, are precisely the urban profiles other vulnerability-generating mechanisms are also most severe in. Based on this finding, it can be concluded that the spatial patterns of the most rapid growth rates for population and urban expansion circa the year 2000 are in direct conflict with vulnerability-reduction efforts to climatic extreme events (Fig 2, 2.4.). This conflict can be expected to worsen under potential future increases in exposure and sensitivity to flooding and cyclones. We suggest that urban areas are particularly sensitive to future increases if their profiles exhibit a) very low current (circa the year 2000) exposure and adaptive capacity (Fig 2, 2.5a), or b) high sensitivity (damage/occurrence ratio) (Fig 2, 2.5b).

Case a) applies to the 51m people in 41 countries (18% of the rapidly urbanizing coastal fringe) constituting the impoverished Maumere profile. Increases in exposure to flooding, e.g. due to climate change, may cause particularly severe managerial problems: The very low occurrence level circa the year 2000 and very low adaptive capacity suggest high damages once exposure increases. This situation is not acting as an early warning signal for potential future increase in damages because of sea-level rise, storm surges, and pluvial and fluvial flooding. Case b) applies to 196m people in 58 countries (38.5% of the rapidly urbanizing coastal fringe) in profiles with low and high income levels—the Bluefields profile, Sittwe and Muisne profiles. Currently, low to moderate occurrence in these profiles already translates into similar damages observed under much higher occurrence in other profiles. This suggests a low capacity for climate-related disaster prevention or post-disaster management. This deficiency is particularly concerning, because ongoing sea-level rise is co-occurring with high values of rapid growth, urban poverty, and low-lying settlement.

### 4.6. Building on government effectiveness and experience provides entry points for vulnerability reduction (Cebu profile)

Generally speaking, it is critical for LICs and MICs is to reduce risks from climate change to urban areas through mindful and integrated strategies for urban land-use planning, channeling settlement, and climate adaptation [96]. In this context, Huq et al. [51] indicate that pro-poor urban climate adaptation policies are needed. These policies are particularly effective under the involvement of households, communities, but also various levels of government [8]. Measures of upgrading informal settlements through collective organization have shown potential to reduce vulnerability to extreme climate events in multiple African and Asian cities [90].

Ideally, jointly enhancing a) the adaptive capacity of flood-sensitive slum populations and b) the effectiveness of government-led reduction of exposure and sensitivity would benefit all flood-vulnerable profiles (Group II and Muisne profiles) (Fig 2, 2.6). A conceivable strategy for reducing the causes of severe exposures is diverting extremely rapid urban expansion from heavily populated, and remaining unpopulated, risk-prone low-lying areas. Likewise, a conceivable strategy for reducing a cause of severe exposures is to protect the limited remaining wetlands from further encroachment. Another effective response is the community-based initiative “Homeless People’s Federation of the Philippines” [90,97]. If pronounced social disparities indicate poor top-down organizational abilities to implement such measures, such as in the Muisne profile, or governments lack resources or political will, collective organization in the affected communities may be a more promising way to reduce vulnerability [21].

### 4.7. Discussion of the method

This study provides the first approximation of the recent situation of coastal vulnerability of urban populations under rapid urbanization. The data sets for the identification of this global typology for coastal urban vulnerability under rapid urbanization are available at [98]. Recently the opportunity for a follow-up study, which employs the most recent data, has emerged.

We conducted our analysis between 2010 and 2015 with the aim of providing the most recent approximation of the current situation. At the time of this analysis, the most recent year for which all of the georeferenced indicator datasets were available was the year approx. 2000. For this common year of reference we used the best available datasets. The common dataset reference to the year 2000 also avoided retro-causality between indicator datasets.

We recognize that new data has become available in recent years for most indicator datasets. For example, the 2009 Global Assessment Report on Disaster Risk Reduction has since superseded the data from Dilley et al. [75] on floods and tropical cyclones by offering a higher resolution and improved modeling of risk frequency and severity [99].Further datasets became available after this analysis was completed. For example, a newer version of the Gridded Population of the World (GPW) v.4 dataset was published in 2016 [100]. However, the global data wetlands prevalence dataset has not been updated to a more recent time frame.

Our results can be interpreted as a missing baseline for commonalities and differences in vulnerability between rapidly urbanizing coastal areas based on the most recent and complete available data at the time of analysis between 2010 and 2015. Overall, we did not redo the analysis with the more recent datasets, because our complete indicator set in a common time frame (2000) supplies a useful near-recent baseline in its own right: First, a baseline to compare to does not exist yet. Second, now that more recent data has become available, the opportunity can be seized to use the same methodology with the latest data. Comparing these results with our baseline would elicit how vulnerability patterns change over time. Follow-up studies can be also based on projected indicator data from an integrated assessment model such as IMAGE. In proof of this concept, Lüdeke et al. [30] have already analyzed changes in the typology of smallholder vulnerability to global environmental change in global drylands using projected data from IMAGE.

The analysis has a few limitations. The first limitation is the specificity of an indicator, which can show a large spread around its cluster center value (i.e. stretched central quartiles, see Fig 5). This means that the cluster membership of a grid cell may not say much about each specific indicator. In this case it is important that the cluster center value of such an indicator does not play a decisive role in the interpretation of the cluster or profile group it belongs to. The second limitation is that our results do not distinctively address specific urban agglomerations, or cities, according to their administrative boundaries, but rather grid cells. The reason for this design is clear: It is not feasible to unambiguously assign all comparable subnational data to rapidly urbanizing coastal cities globally. The third limitation is the use of national-level data when subnational data was either unavailable (e.g. for government effectiveness), or unfeasible (for GDP per cap), for capturing local or regional vulnerability-generating phenomena in urban areas. At the same time, national level GDP per cap differentiates between LICs, MICs, and HICs. This can help indicate the resources a national economy can mobilize for vulnerability reduction, e.g. for climate change adaptation [90]. We ruled out using the subnational dataset of ‘‘gross cell product” per cap [101], because numerous coastal countries with rapid growth lack the data.

## 5. Conclusions

We developed and discussed a global spatially explicit systematization of typical mechanisms that generate coastal urban vulnerability under rapid urbanization. The systematization is for the year 2000. The results show that typical combinations of these links exist, and where they occur, regardless of whether a case study exists in this particular location or not. On a global scale, the results advance the knowledge of links between typical socio-ecological problem structures and vulnerabilities in coastal urban areas under rapid urbanization in LICs and MICs. On a regional to a more local level the results show that local vulnerability-generating mechanisms recurrently formulated in city-based case studies can be robustly identified across coastal regions.

Generally speaking, income and government effectiveness decrease with the rate of urban population growth, while urban poverty increases. Furthermore, we underlined the criticality of flood occurrence and damage in shaping coastal urban vulnerability under rapid growth in LMICs. Cyclone occurrence and damage, and government effectiveness, are also important. There are huge asymmetries between profiles regarding vulnerability and how they are positioned to deal with and reduce vulnerabilities. This is the case in many LDCs along Western and Eastern African tropical coasts, for example. Spatial patterns of the most rapid growth rates for population (two profiles) and urban expansion (three profiles) are in direct conflict with vulnerability-reduction efforts to climatic extreme events for 247m people in 75 countries (18% of the rapidly urbanizing coastal fringe). This conflict can be expected to worsen under potential future increases in exposure and sensitivity to flooding and cyclones. In order to reduce risk, which is the critical issue for climate adaptation in LICs and MICs [96], this calls for a mindful, integrated, and effective approach of urban land-use planning together with climate adaptation.

On a generic level this typological approach may facilitate learning and scaling up successful place-based vulnerability reduction in areas sharing the same profile, i.e. areas with similar socio-ecological problem structures. Thereby the hypothesis is that a similar problem structure suggests comparable intervention options, i.e. that the typology of adaptation needs may be a typology of adaptation responses. This hypothesis has been corroborated by Kok et al. [25] and Sietz et al. [18], yet needs to be explored further in an urban setting. Under limited funds, time, and human resources available for vulnerability reduction in our time this could help the urgently needed replication, and upscaling of vulnerability-reducing measures that were successful in a certain urban context, across specific urban profiles.

The urban profiles identified in this study can also be useful for increasing the understanding of other societal outcomes that are conceivably related to the coastal vulnerability of urban populations, such as conflict incidence. A study based on the same methodology applied here has already increased the understanding of how violent conflict incidence and distribution is linked to typical socio-ecological problem structures in a global typology of vulnerability in drylands [102].

Follow-up studies should analyze changes in urban profiles relative to the baseline of this study. We recommend prioritizing the use of the most recent common time frame that all required datasets are available in as opposed to simply including the most recent, best available data. Furthermore, studies should analyze the dynamics of the urban vulnerability profiles, and what this means for future risk management, using projected socio-economic and environmental change data from integrated assessment models. Using the same methodology, Lüdeke et al. [30] have already conducted such a study for detecting and understanding change in profiles of vulnerability in drylands.

## Supporting information

S1 Fig Consistency measure, plotted against the cluster number for our 11-dim data set. (TIF)
