# Supplementary figures and images for: Typology of coastal urban vulnerability under rapid urbanization
Source: PLoS One. 2020 Jan 31;15(1):e0220936. doi: 10.1371/journal.pone.0220936 (PMC6993965; doi:10.1371/journal.pone.0220936)

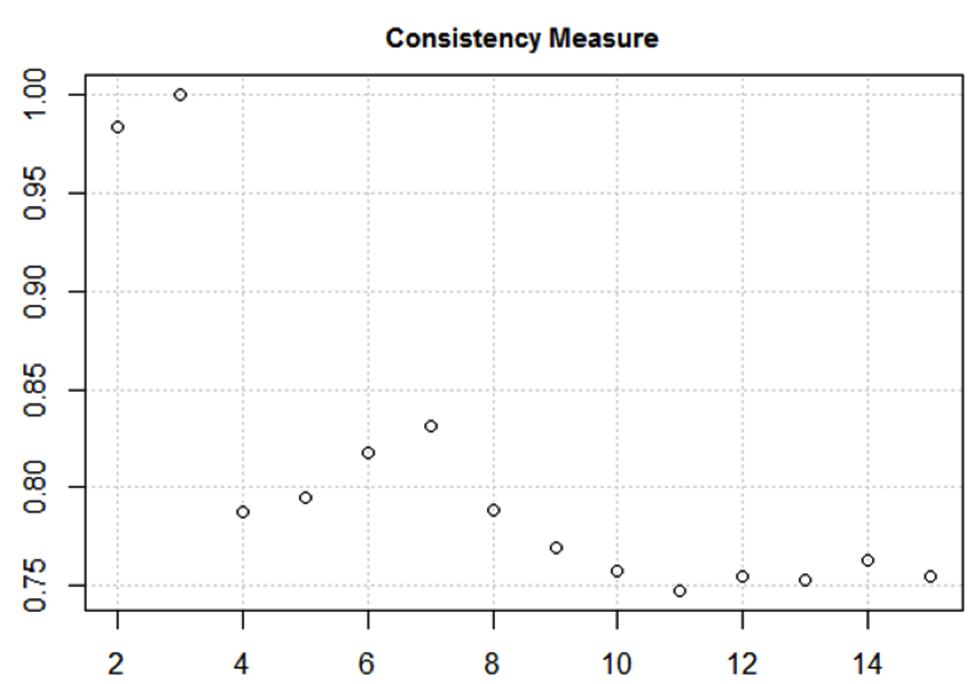

Supplement: S1 Fig — (TIF) [file pone.0220936.s001.tif]
